# Exploring spillover effects following surgical de-implementation: an observational evaluation of primary care, referrals, and time to surgical intervention following reductions in the use of tonsillectomy and Dupuytren’s contracture

**DOI:** 10.1186/s43058-026-00857-7

**Published:** 2026-02-12

**Authors:** Joel Glynn, Timothy Jones, Sam Creavin, Carmel Conefrey, Jenny Falco, Nicola Farrar, Mike Bell, Jane Blazeby, Christopher Burton, Jenny L. Donovan, Andy Gibson, Angus McNair, Josie Morley, Amanda Owen-Smith, Ellen Rule, Gail Thornton, Victoria Tucker, Iestyn Williams, William Hollingworth, Leila Rooshenas

**Affiliations:** 1 Bristol Medical School, Population Health Sciences, University of Bristol, 1-5 Whitleladies Road, Bristol, BS8 1NU England; 2 Musculoskeletal Research Unit, Bristol Medical School, Translational Health Sciences, Bristol, England; 3 Public Contributor, Bristol, England; 4 National Institute for Health Research Biomedical Research Centre (NIHR BRC), University Hospitals Bristol and Weston NHS Foundation Trust and University of Bristol, Bristol, England; 5 Bristol Medical School, Population Health Sciences, Bristol Centre for Surgical Research, University of Bristol, Bristol, England; 6 School of Allied and Public Health Professions, Canterbury Christ Church University, Canterbury, England; 7 Department of Health and Social Sciences, University of the West of England, Bristol, England; 8 NHS Gloucestershire Integrated Care Board (ICB), Gloucester, England; 9 NHS Bristol North Somerset and South Gloucestershire Integrated Care Board (ICB), Bristol, England; 10 Health Services Management Centre, University of Birmingham, Birmingham, England

**Keywords:** De-adoption, De-implementation, Tonsillitis, Dupuytren’s Contracture, Surgery, Primary Care

## Abstract

**Background:**

Reducing the use of low-value surgery is important to maintain effective, safe and financially sustainable health systems. However, following de-implementation there are inevitably wider impacts for health systems beyond the targeted reduction in surgery, which have hitherto not been explored. Here we describe the spillover effects following the reduction in the use of two surgical procedures; tonsillectomy and Dupuytren’s contracture release (DCR) identified in 2019 by the Evidence Based Interventions (EBI) programme a de-implementation initiative in England.

**Methods:**

This longitudinal observational study used linked primary (Clinical Practice Research Datalink) and secondary (hospital episode statistics) care electronic health records from 1st April 2016 to 29th February 2020 to identify care for patients presenting with symptoms of tonsillitis or Dupuytren’s contracture (DC). Outcome measures include GP visits; related prescriptions; outpatient appointments, referral to secondary care and surgery. Differences were explored between cohorts of patients seeking care for tonsillitis or DC before and after EBI guidelines. Using a two-part Generalised Linear Model (GLM), we explored changes in likelihood of surgery and the time-to-surgery (if it occurred) before and after EBI guidelines.

**Results:**

Repeat GP visits for tonsillitis increased by 540 per 10,000 patients-per-year post-EBI, but remained stable for DC. Antibiotic prescriptions for tonsillitis fell, and outpatient appointments remained stable for both conditions.

The likelihood of surgery reduced for both conditions post-EBI with an odds ratio of 0.75 {95%CI 0.71, 0.79} and 0.88 {95%CI 0.81, 0.95}), with a shorter time-to-tonsillectomy of -5.2 days (95%CI {-9.5 days to -1 day}). Reduction in time-to-DCR was less evident (-2.2 days CI {95%-7.1 to + 3}) but should be considered in the context of nationally increasing elective waiting time trends.

**Conclusions:**

Reductions in both surgical procedures were associated with small, but potentially important, changes to primary care utilisation and specialist treatment. Policy makers should identify potential spillovers from de-implementation, design policies to mitigate negative effects, and monitor these wider impacts alongside the direct influence on the targeted procedure rates.

**Supplementary Information:**

The online version contains supplementary material available at 10.1186/s43058-026-00857-7.

Contributions to the literature

- De-implementation of low-value surgery has potential spillover effects on individual patient care and the wider healthcare system which are rarely measured.
- In the context of the English Evidence Based Interventions national de-implementation programme, we observed reductions in tonsillectomy and Dupuytren’s contracture release surgical procedures.
- We found potentially important, positive (e.g. reduced time-to-surgery) and negative (increased repeat GP consultations) spillovers associated with reductions in the use of surgery.
- We advocate for the identification and evaluation of spillovers when evaluating de-implementation initiatives, allowing the capture of wider benefits, and helping avoid the reallocation of scarce healthcare resources to other low-value care.

## Introduction

Internationally health systems typically aim to provide timely, high-quality care that is accessible to the whole population from limited budgets. Achieving these aims have been increasingly challenging for the National Health Service (NHS) in England over the last decade as growth in the healthcare budget has been insufficient to meet rising demand [1, 2]. One approach to reducing pressures on health care budgets is to identify and de-implement existing ‘low-value’ healthcare.

‘De-implementation’ is the process of stopping or reducing a clinical practice that has become embedded in routine healthcare [3]. De-implementation of a health intervention may result in its complete removal from practice, or more commonly the restriction of its use to groups of patients based on clinical criteria such as severity, number of episodes and impacts on quality of life. Low value care refers to any healthcare where the risks and costs of its use outweigh the benefits to certain patients [4]. This comprises care that is either harmful, low in efficacy, or not cost-effective in a given setting. Low-value care has significant opportunity costs, displacing or delaying treatments that more meaningfully improve patient health, as well as in many cases subjecting patients to unnecessary risk. Given increasing healthcare demand [5] and the perpetual development of effective novel interventions, de-adoption of low-value care is vital to sustaining affordable health services, but changing entrenched practices is difficult [6–8].

In 2018 NHS England launched the ‘Evidence-Based Interventions’ (EBI) Programme [9]. The EBI programme is a national de-implementation programme focusing predominantly on surgical interventions and diagnostic testing. It aimed to improve care for the population through optimising the use of finite resources, reducing the risk of harm to patients, and minimising unwarranted variation in service provision across the country [9]. To date, the programme has published four waves of recommendations, each specifying access criteria for interventions that are considered inappropriate for some patients, and therefore over-used in the NHS [10]. The first set of recommendations was published in April 2019, identifying 17 surgical procedures for de-implementation [11]. Local healthcare budget holders, now called Integrated Care Boards (ICBs, previously clinical commissioning groups, CCGs), were expected to stop funding four ‘category 1’ procedures considered clinically ineffective. Category 1 procedures should only be funded through individual funding requests in exceptional circumstances [12]. In addition, CCGs should restrict funding for 13 ‘category 2’ procedures to patients who meet specific clinical criteria published by the EBI programme. A 12-month target was set for CCGs to reduce use of category 1 procedures to nearly zero. For category 2 procedures, the goal was to reduce use to match the rate seen in the lowest 25% of ICBs (after adjusting for age and sex differences in their populations).

There is increasing awareness that de-implementation may have both intended and unintended consequences for patients and the NHS that go beyond reduction in the target treatment. These are referred to as ‘spillover effects’. These effects occur as changes are made within one area of a complex and interrelated healthcare system [13]. Spillover effects can both positively or negatively impact patient wellbeing and the efficient use of limited resources. In this case, surgical de-implementation may lead to changes in the type, amount and timeliness of care patients receive elsewhere in the health system. It is important to capture these changes so that policy makers and patients can fully understand the overall impact of de-implementation. However, spillover effects are not commonly measured.

This paper forms part of the OLIVIA study, a larger mixed methods evaluation of the EBI programme in the NHS [14]. Previous and forthcoming work has assessed the impact of the programme on procedure rates, geographic variation and qualitatively captured views from patients, clinicians and commissioners [7]. In this paper we use primary care data, available from the clinical practice research datalink (CPRD), linked to Hospital Episode Statistics (HES) inpatient and outpatient data, to explore how increasing restrictions on access to NHS-funded surgery might cause ‘spillover’ effects on primary care treatment and referral pathways [13].

We selected two case study conditions: tonsillitis and Dupuytren’s contracture of the hand. Both tonsillectomy and Dupuytren’s contracture release (DCR) were included as ‘category 2’ procedures in wave one of the EBI programme. These case study procedures were selected based on several factors, including: applicability to different populations (i.e. tonsillectomy was predominantly relevant to children and young adults, while DCR tended to be more prevalent in older adults); different clinical specialties (i.e. ENT versus hand surgery/orthopaedics); and uncertain and evolving evidence about the value of surgery (i.e. randomised controlled trials were in progress for tonsillectomy [7, 15] and DCR [16, 17]). For both, procedure rates had been generally declining over time in the NHS for several years [7, 15].

### Case study condition one: tonsillitis

Recurrent sore throat or tonsillitis is a common condition affecting adults and children with a significant impact on quality of life, including ability to attend school or work. Most cases are treated conservatively, including the use of common pain medications, and, for bacterial tonsillitis, antibiotics. A short course of steroids (e.g. dexamethasone) is also sometimes prescribed. The EBI programme set criteria (e.g. based on frequency and severity of symptoms) to target surgery at patients believed to be most likely to benefit [18]. The EBI programme estimated 7,454 tonsillectomies could be avoided each year if the criteria were followed.

### Case study condition two: Dupuytren’s contracture

Dupuytren’s contracture occurs when fibrous bands in the palm of the hand progressively draw.

the finger(s) towards the palm and prevent them from straightening fully. Treatment includes Dupuytren Contracture Release (DCR) surgery (e.g. fasciectomy; dermo-fasciectomy; or needle fasciotomy), and hand therapy [19, 20]. The EBI programme set criteria for surgery based on the degree of contracture (> 20° or > 30° depending on finger joint) and functional impact [21]. The EBI programme estimated 4,113 DCRs could be avoided each year if the EBI criteria were followed.

Our objective was to identify the potential spillover effects of EBI de-implementation guidance on patients care and resource use following a General Practitioner (GP) visit for one of the case study conditions. Specifically, we aimed to 1) measure differences in the use of primary care, secondary care referral and relevant medications and 2) explore whether the likelihood and time to surgery differed before and after the EBI guidance.

## Methods

### Study design

We present a repeated cross-sectional observational study with longitudinal follow up, using routinely collected administrative data on patients registered with UK GP practices from 1 st April 2016 to 29th February 2020. It is reported according to the REporting of studies Conducted using Observational Routinely Collected Health Data (RECORD) [22] guidelines.

### Data sources

Data was received from CPRD Aurum (May 2022 snapshot). CPRD Aurum captures data from GP practices using the EMIS electronic health record software. At the time of extract CPRD Aurum included 13.3 million current patients, which is approximately 19.8% of the UK population with 93% eligible for linkage to hospital datasets [23]. Patients are broadly representative of the general population in England regarding age, sex, deprivation, ethnicity, and geographical spread [24]. NHS England provides patient level data linkage to episode-level data from the English hospital episode statistics (HES) admitted patient care (APC) and outpatient (OP) datasets [25, 26]. Area level socioeconomic status was also derived using the Index of Multiple Deprivation (IMD) 2019, based on patient residential postcode area.

### Study population

We requested full medical records for all patients included in CPRD Aurum with linked HES data if they had at least one GP visit with a recorded symptom code indicating tonsillitis (any age) or Dupuytren’s contracture (adults aged 18 +), within either of the two time period cohorts detailed below, according to the medical code lists capture in CPRD.

1. Cohort one: all patients with a relevant GP visit between 1 st April 2016 to 28th February 2017 (at least two years before EBI guidance was published);
2. Cohort two: all patients with a relevant GP visit between 1 st April 2019 to 29th February 2020 (after EBI guidance).

Medical codes were determined using the CPRD ‘Code Browser’ version 3.0.0 [27] applying key words associated with the two conditions. Codes were then refined through discussion with a General Practitioner (SC), full code lists are available in Supplementary Materials S1. A single ‘index’ GP visit was defined for each patient as the earliest visit with the relevant symptoms recorded within the study period. Analysis was completed separately for each condition.

### Variables

We intentionally excluded any healthcare use after February 29th 2020 for cohort two given significant restrictions on elective healthcare in England at the onset of COVID-19 pandemic. To maintain consistent follow-up windows in each cohort, we also excluded healthcare after February 28th 2017 for cohort one. Each patient had different durations of follow-up, which represents the time between their index GP visit, defined above, and the earliest of; the end of February (2017 for cohort one and 2020 for cohort 2), the end of registration at the practice, or death. The cohorts were separated by a two-year period, allowing two years of treatment to occur prior to the publication of EBI guidance in 2019. Consequently, we are confident healthcare decisions in the pre-EBI cohort were not influenced by EBI guidelines.

For all patients we extracted demographic information on age, sex, ethnicity (based on ONS classifications [28]), and deprivation quintiles (1 = least deprived).

All GP visits (for symptoms of tonsillitis or Dupuytren’s contracture) were extracted that occurred after the patients index GP visit. Given the index (first) GP visit within our study period may not be the patients first GP visit for their condition; we captured the number of GP visits for symptoms of tonsillitis or Dupuytren’s contracture that occurred in the previous year (i.e. 2015/16 and 2018/19).

Using linked HES Outpatient and Admitted Patient Care data we extracted hospital outpatient appointments for the relevant specialty (Ear Nose and Throat (ENT) for tonsillitis, trauma/orthopaedics or plastic surgery for Dupuytren’s contracture), and all hospital admissions with tonsillectomy or DCR recorded as the primary procedure.

For tonsillitis patients we additionally captured all GP prescriptions for both antibiotics and dexamethasone. No prescription we collected for Dupuytren’s contracture patients.

### Missing data

CPRD is a large dataset with minimal (< 0.2%) missing data for age, sex and area-based deprivation. Therefore, patients with missing values for these variables were excluded from regression analyses. Ethnicity is more frequently recorded as unknown or missing, in 2023 81.7% of CPRD linked to HES had a code for ethnicity [29]. The dataset is considered to have suitable representation of ethnicities compared to the UK general population, with slight over representation of ethnic minority groups [29]. For our primary analyses we coded missing ethnicity as ‘unknown/missing’. To explore the possible impact of this approach, we conducted sensitivity analyses excluding patients with unknown or missing ethnicity data. Where there were no recorded GP visits, hospital/outpatient visits, or prescriptions after the index GP visit, we assumed there were no subsequent healthcare interactions for this patient, consistent with CPRD/HES data capture processes.

### Statistical analysis

Separately for tonsillitis and Dupuytren’s contracture, we compared the use of healthcare before and after EBI. Specifically, we compared GP visits, prescribing of antibiotics or dexamethasone (tonsillitis only), outpatient appointments, and surgery following the index GP visit. In those that had surgery, we also compared the number of days between the index GP visit and surgery. In unadjusted analyses, we present mean differences and bootstrapped 95% confidence intervals (for count variables), mean difference in days until surgery and 95% confidence intervals [30], and the difference in proportions and 95% confidence intervals of patients receiving surgery.

### Two-part model

We explore changes in the likelihood of surgery and days until surgery for tonsillitis and Dupuytren’s contracture. Given that only a subset of patients go on to have DCR or tonsillectomy surgery, a two-part model was chosen which estimates the likelihood of surgery in all patients and then estimates time to surgery in the subset that go on to have it. The first part of the regression model used logistic regression to explore the odds of patients receiving surgery before and after EBI. The second part of the model used a Generalised Linear Model (gamma distribution, log link) to explore surgical waiting times, for those who receive surgery. We included a cohort indicator (Cohort One/Cohort Two), sex, age, related GP visits in the previous year, IMD deprivation quintiles and ethnicity as independent variables in the two-part model. Using the Stata *margins* command, we transformed the regression output to the raw scale (i.e. days until surgery) to explore the potential impact of the EBI guidelines on surgical waiting times, holding all other covariates at their means. All analyses were conducted in Stata18.

## Results

**Fig. 1:**
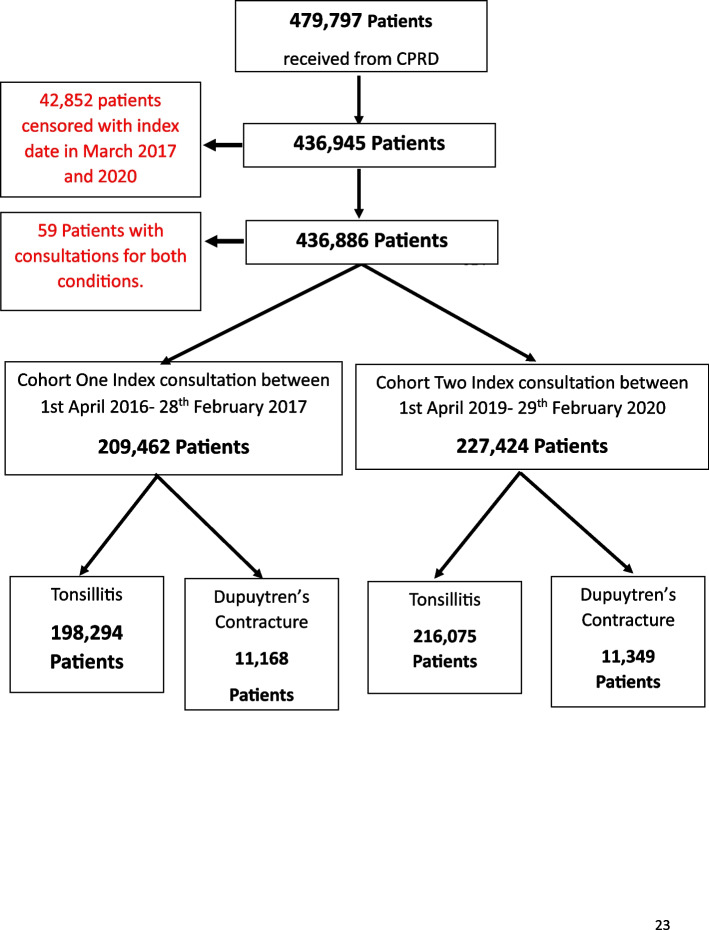
Data flow diagram

We received linked data for 479,797 patients from CPRD, we censored 42,852 with an index GP visit in March 2017 or March 2020 (Fig. 1). An additional 59 patients were dropped as they had consultations recorded for both tonsillitis and Dupuytren’s contracture. Very few patients had missing age [59 patients, 0.01%] and sex [9 patients, < 0.01%]. Our analysis dataset included a total of 414,369 individuals who attended a GP with tonsillitis symptoms and 22,517 attending with Dupuytren’s Contracture symptoms (Table 1).

**Table 1:** Descriptive information for people with a GP visit for either tonsillitis or Dupuytren’s Contracture: 1 st April 2016 to 28th February 2017 vs April 2019 to 29th February 2020

	**Tonsillitis**		**Dupuytren's**	
	**Cohort one 2016/17**	**Cohort two 2019/20**	**Cohort one 2016/17**	**Cohort two 2019/20**
Population	198,294 (100%)	216,075 (100%)	11,168 (100%)	11,349 (100%)
Sex				
*Men*	80,135 (40.4%)	87,353 (40.4%)	7708 (69%)	7800 (68.7%)
*Women*	118,157 (59.6%)	128,715 (59.6%)	3460 (31%)	3549 (31.3%)
*Indeterminate*	2 (0%)	7 (0%)	(0%)	(0%)
Age Group				
*0–9*	68,408 (34.5%)	76,074 (35.2%)	(0%)	(0%)
10–19	40,750 (20.6%)	44,256 (20.5%)	1 (0%)	4 (0%)
*20–29*	39,166 (19.8%)	43,221 (20%)	63 (0.6%)	35 (0.3%)
*30–39*	28,599 (14.4%)	30,734 (14.2%)	180 (1.6%)	197 (1.7%)
*40–49*	12,673 (6.4%)	12,518 (5.8%)	794 (7.1%)	631 (5.6%)
*50–59*	5524 (2.8%)	5860 (2.7%)	2473 (22.1%)	2451 (21.6%)
*60–69*	2141 (1.1%)	2271 (1.1%)	3886 (34.8%)	3796 (33.4%)
*70–79*	783 (0.4%)	840 (0.4%)	2804 (25.1%)	3122 (27.5%)
*80* +	250 (0.1%)	301 (0.1%)	967 (8.7%)	1113 (9.8%)
Deprivation				
*1—least deprived*	35,749 (18%)	38,489 (17.8%)	2978 (26.7%)	3017 (26.6%)
*2*	36,312 (18.3%)	39,999 (18.5%)	2680 (24%)	2798 (24.7%)
*3*	36,730 (18.5%)	39,956 (18.5%)	2160 (19.3%)	2233 (19.7%)
*4*	41,919 (21.1%)	45,575 (21.1%)	1786 (16%)	1792 (15.8%)
*5—most deprived*	47,420 (23.9%)	51,887 (24%)	1555 (13.9%)	1500 (13.2%)
*missing*	164 (0.1%)	169 (0.1%)	9 (0.1%)	9 (0.1%)
Ethnicity				
*Asian, Asian British, Asian Welsh*	14,490 (7.3%)	14,913 (6.9%)	133 (1.2%)	138 (1.2%)
*Black, Black British, Black Welsh, Caribbean or African*	6321 (3.2%)	6130 (2.8%)	59 (0.5%)	77 (0.7%)
*Mixed, Multiple*	4255 (2.1%)	5095 (2.4%)	23 (0.2%)	28 (0.2%)
*White*	135,804 (68.5%)	140,153 (64.9%)	10,107 (90.5%)	10,050 (88.6%)
*Other*	4843 (2.4%)	5129 (2.4%)	76 (0.7%)	93 (0.8%)
*Unknown/Missing*	32,581 (16.4%)	44,655 (20.7%)	770 (6.9%)	963 (8.5%)
GP Practice visit for symptoms of tonsillitis/Dupuytren’s (1 year before)				
*0*	176,512 (89%)	193,215 (89.4%)	10,029 (89.8%)	10,067 (88.7%)
*1*	15,264 (7.7%)	15,922 (7.4%)	792 (7.1%)	897 (7.9%)
*2*	3974 (2%)	4373 (2%)	217 (1.9%)	236 (2.1%)
*3* +	2544 (1.3%)	2565 (1.2%)	130 (1.2%)	149 (1.3%)
Antibiotic Prescriptions (1 year before)				
*0*	126,915 (64%)	147,659 (68.3%)		
*1* +	71,379 (36%)	68,416 (31.7%)		
Dexamethazone Prescriptions (1 year before)				
*0*	196,974 (99.3%)	214,460 (99.3%)		
*1* +	1320 (0.7%)	1615 (0.7%)		

### Comparison of patient cohorts at the index GP visit

- *Tonsillitis*

198,294 patients attended the GP with tonsillitis symptoms in Cohort One. This increased to 216,075 in Cohort Two. The characteristics of patients with tonsillitis symptoms were similar between cohorts (Table 1). These patients were predominantly white and under 30 years of age. There were slightly more females and more patients living in areas associated with greater rather than less deprivation. Approximately 10% had visited their GP with symptoms of tonsillitis in the year before their index GP visit. Greater than 30% had been prescribed an antibiotic. The mean duration of follow-up was 166 days in Cohort One and 159 days in the Cohort Two.

b. *Dupuytren’s Contracture*

11,168 and 11,349 patients attended the GP with evidence of Dupuytren’s Contracture in Cohorts One and Two respectively. The profile of patients seeking GP care for symptoms of Dupuytren’s Contracture was similar between cohorts (Table 1). These patients were predominantly white (90%), male (69%), and living in less deprived areas of the UK. Approximately 10% had visited their GP in the year before their index GP visit. The number of patients attending their GP was stable between cohorts. The mean duration of follow-up was 177 days for both cohorts.

### The Use of healthcare after the index GP visit

- *Tonsillitis*

Repeat consultations were higher in Cohort Two (mean difference 0.013, 95% CI {0.009 to 0.018}, Table 2. This is an increase of 560 appointments per 100,000 patients. The use of antibiotics was moderately less common in Cohort Two (mean difference −0.029, 95%CI {−0.034, −0.024}; Table 2). Very few patients had either a dexamethasone prescriptions (0.8%) or outpatient appointments (< 5%) across both time periods.

**Table 2:** Care received after^1^ index GP visit for patients with symptoms of tonsillitis

	**Cohort one** **2016/17** **Mean (95% CI)**	**Cohort two** **2019/20** **Mean (95% CI)**	**Difference (95% CI)**
GP Visits (tonsillitis)	0.236 (0.233, 0.239)	0.249 (0.246, 0.252)	0.013 (0.009, 0.018)
Antibiotic Prescriptions	0.409 (0.405, 0.413)	0.38 (0.376, 0.383)	−0.029 (−0.034, −0.024)
Dexamethasone Prescriptions	0.001 (0.001, 0.001)	0.002 (0.002, 0.003)	0.001 (0.001, 0.002)
ENT OP Appointments	0.073 (0.071, 0.075)	0.073 (0.071, 0.075)	0 (−0.002, 0.002)
Tonsillectomy (%)	1.58% (1.52%, 1.63%)	1.22% (1.18%, 1.27%)	−0.36% (−0.39%, −0.32%)
Days until surgery^2^	127.6 (124.8, 130.4)	122.4 (119.4, 125.4)	−5.2 (−9.5, −1)

^1^Until 28th February 2017 for cohort one or until 29th February 2020 for cohort two

^2^For those that have a tonsillectomy

Less than 2% of patients went on to have a tonsillectomy within our analyses, however this was less likely in cohort two (mean percentage difference −0.36%, 95% CI {−0.39% to −0.32%}; Table 2). Time to surgery was approximately 4 months from their index GP visit, but was 5 days shorter in Cohort Two (mean difference −5.2 days; 95% CI {−9.5, −1 days}; Table 2).

b. *Dupuytren’s Contracture*

Repeat GP consultations were similar in both cohorts (mean difference in consultations 0.013, 95% CI:{ −0.006 to + 0.031}, Table 3). However, represented a point estimate of approximately 268 more appointments per 100,000 patients in Cohort Two. Unlike tonsillitis, outpatient appointments were common for Dupuytren’s contracture with an average of 1 per-patient, with an increase of 860 appointments per 100,000 patients (mean difference 0.045, 95%CI {−0.011, + 0.101}, Table 3), however uncertain due to overlapping confidence intervals.

**Table 3:** Care received after^1^ index GP visit for patients with symptoms of Dupuytren’s Contracture

	**Cohort one** **2016/17** **Mean (95% CI)**	**Cohort two** **2019/20** **Mean (95% CI)**	**Difference (95% CI)**
GP Visits (Dupuytren's)	0.258 (0.245, 0.271)	0.271 (0.258, 0.284)	0.013 (−0.006, 0.031)
Orthopaedics/Plastic Surgery OP Appointments	1.093 (1.054, 1.131)	1.138 (1.1, 1.176)	0.045 (−0.011, 0.101)
DCR (%)	13.9% (13.2%, 14.5%)	12.3% (11.7%, 12.9%)	−1.6% (−2%, −1.1%)
Days until surgery^2^	113.8 (110.4, 117.2)	111.6 (107.9, 115.3)	−2.2 (−7.3, 3)

^1^Until 28th February 2017 for cohort one or until 29th February 2020 for cohort two

^2^For those that have surgery (DCR)

Approximately 13% of patients had a DCR procedure during the follow up period. The proportion having a procedure was lower in Cohort Two (mean difference −1.6%, 95% CI {−2% to −1.1%}; Table 3). Time to surgery was approximately 4 months from their index GP visit, but was 2 days shorter in Cohort Two (mean difference −2.2 days, 95% CI {−7.3 days to 3days}; Table 3).

### Factors associated with surgery and wait time for surgery* (Two-part Model)*

The results from the two-part model are presented in Tables 4 and 5. 342 tonsillitis patients and 18 Dupuytren’s contracture patients (0.08% of total patients) were excluded from the regression model due to missing data on age, sex or deprivation.

**Table 4:** Odds of having tonsillectomy or DCR surgery following an index GP visit

	Tonsillectomy Odds Ratio (95% CI), *n* = 414,027	DCR Odds Ratio (95% CI), *n* = 22,499
Cohort		
*One (2016/17)*	1	1
*Two (2019/20)*	0.77 (0.73, 0.81)	0.88 (0.81, 0.96)
Sex		
*male*	1	1
*female*	1.09 (1.03, 1.16)	0.58 (0.53, 0.64)
Age (years)	1.02 (1.01, 1.02)	0.99 (0.98, 0.99)
GP Visits (last year)		
*0*	1	1
*1*	5.31 (4.96, 5.69)	3.41 (3.04, 3.82)
*2*	12.29 (11.29, 13.38)	4.42 (3.62, 5.39)
*3* +	32.45 (30.01, 35.1)	2.99 (2.29, 3.91)
IMD 2019		
*1—least deprived*	1	1
*2*	1.14 (1.04, 1.25)	1.06 (0.95, 1.18)
*3*	1.16 (1.06, 1.27)	1.09 (0.97, 1.23)
*4*	1.22 (1.11, 1.33)	1.15 (1.02, 1.3)
*5—most deprived*	1.24 (1.14, 1.35)	0.9 (0.78, 1.03)
Ethnicity		
white	1	1
*Asian*	0.76 (0.69, 0.85)	0.43 (0.26, 0.7)
*black*	0.88 (0.74, 1.03)	0.5 (0.26, 0.96)
*mixed*	1.07 (0.92, 1.24)	0.5 (0.15, 1.62)
*other*	0.89 (0.75, 1.05)	0.55 (0.31, 0.98)
*unknown/missing*	0.11 (0.1, 0.13)	0.43 (0.35, 0.52)

**Table 5:** Days to surgery for people having tonsillectomy or DCR following an index GP visit

	**Tonsillectomy Ratio (95% CI)**, ***n* = 5761**	**DCR Ratio (95% CI)**, ***n* = 2942**
Cohort		
*One (2016/17)*	1	1
*Two (2019/20)*	0.95 (0.92, 0.98)	0.99 (0.94, 1.04)
Sex		
*male*	1	1
*female*	1.03 (0.99, 1.07)	0.99 (0.93, 1.05)
Age (years)	1 (1, 1.01)	1.002 (1, 1.005)
GP Consultations (last year)		
*0*	1	1
*1*	1.07 (1.03, 1.12)	0.76 (0.71, 0.81)
*2*	1.04 (0.99, 1.1)	0.57 (0.51, 0.64)
*3* +	0.97 (0.92, 1.01)	0.56 (0.48, 0.65)
IMD 2019		
*1—least deprived*	1	1
*2*	1.01 (0.95, 1.07)	0.98 (0.92, 1.05)
*3*	0.98 (0.93, 1.04)	0.97 (0.9, 1.04)
*4*	0.98 (0.93, 1.04)	0.95 (0.88, 1.02)
*5—most deprived*	0.99 (0.94, 1.05)	1.01 (0.93, 1.1)
Ethnicity		
*white*	1	1
*Asian*	0.97 (0.91, 1.04)	0.99 (0.72, 1.36)
*black*	0.98 (0.88, 1.09)	0.54 (0.36, 0.82)
*mixed*	0.98 (0.89, 1.08)	1.32 (0.62, 2.82)
*other*	0.99 (0.89, 1.11)	1.02 (0.71, 1.46)
*unknown/missing*	0.99 (0.88, 1.11)	1.02 (0.9, 1.16)

- *Tonsillitis*

The number of GP consultations for symptoms of tonsillitis in the previous year was very strongly associated with the odds of having tonsillectomy. Older patients, females and patients living in more deprived areas were more likely to have surgery (Table 4). Patients with 3 or more consultations recorded in the GP record had 32.45 times (95% CI {33.5 to 39.12}; Table 4) greater odds of having tonsillectomy than without a recorded GP consultation. Patients in cohort two were less likely to have surgery (odds ratio 0.77; 95% CI {0.73 to 0.81}; Table 4). There was also evidence of non-white or mixed ethnicities being less likely to have tonsillectomy (Table 4). These findings were consistent in the sensitivity analysis excluding patients with unknown or missing ethnicity (Table S1).

When adjusting for patient characteristics the GLM model predicted those who had a tonsillectomy,

waited fewer days for their procedure (0.95 (95%CI {0.92, 0.98}; Table 5). This amounted to a mean of approximately 127.9 (125.0 to 130.9) days and 121.6 (118.5 to 124.7) days between their index GP visit and surgery in Cohort One and Two, respectively. There was no association between the number of GP consultations in the preceding year and waiting time for surgery (Table 5). There was no evidence that ethnicity is associated with waiting time for tonsillectomy in either the primary or sensitivity analyses (Table 5, Table S2 respectively).

b. *Dupuytren’s contracture*

Patients with more GP consultations were more likely to have surgery. Those with 3 or more consultations recorded in the GP records had 2.99 (95%; CI {2.29 to 3.91}; Table 4) times greater odds of having DCR than those who had no consultations. Younger patients and males were also more likely to have surgery (Table 4). Patients in Cohort Two were less likely to have surgery (odds ratio 0.88; 95% CI {0.81 to 0.96}; Table 4). There is evidence that non-white ethnicities were less likely to have a DCR procedure (Table 4). These findings were consistent in the sensitivity analysis excluding patients with unknown or missing ethnicity (Table S1).

When adjusting for patient characteristics, the GLM model predicted waiting times for surgery were marginally shorter in Cohort Two (0.99, 95% {CI: 0.94,1.04}; Table 5). This amounted to a mean of approximately 111.7 days (108.0 to 115.4) days and 110.6 (106.7 to 114.4) days to surgery in Cohort One and Two respectively. Unlike the tonsillitis patients, there was a very strong association between a number of GP consultations in the previous year and a shorter wait for surgery (Table 5). We observe differences in time to surgery by ethnicity, with patients with black ethnicities being associated with a quicker time to surgery (0.54, 95%CI {0.36,0.82}), these result hold in the complete case results (Table S2).

## Discussion

### Principal findings

Reduction in tonsillectomy and DCR procedures observed between 2016 and 2020 [7] was associated with small, but potentially important, changes to primary care utilisation and patient care. Spillover effects were more pronounced in the tonsillectomy cohort, specifically, the number of primary care re-consultations increased for patients with tonsillitis and there was a reduction in antibiotic prescriptions, despite more consultations occurring. Among those who did get tonsillectomy the time between index GP visit and surgery fell by 5 days. The reduction in DCR surgery however did not significantly increase GP or outpatient appointments. Yet time to surgery in those who had it also did not increase despite nationally increasing waiting times for elective surgery.

Some of these findings might represent positive spillovers, such as the reduction in time-to-surgery for those most likely to benefit. Yet increases in care elsewhere, such as additional GP consultations could be considered low-value use of GP time if purely to document episodes of tonsillitis cases to meet thresholds for surgery. Without the evaluation of potential spillovers alongside de-implementation policy, it is unclear whether successful de-implementation of surgery will result in more or less cost-effective care as resources are reallocated and patient care changes.

### Comparison with other literature and data

The observational nature of our study and the lack of control groups mean that our findings need to be viewed in the context of general trends in healthcare. Firstly, the decrease in antibiotic use observed in the tonsillitis Cohort Two likely reflects decreasing national prescribing trends rather than any spillover effect of the decreased access to tonsillectomy [31]. Secondly, national primary care consultation numbers remained relatively constant across our study period, suggesting that the observed increase in re-consultations for tonsillitis could be a genuine spillover effect [32]. Finally, our finding of a reduction in wait-times between a patients index GP visit and surgery for tonsillectomy and no increase for DCR, should be viewed in the context of NHS-wide data showing large increases in waiting times for elective hospital care over the same time period [1]. Average waiting times for all elective surgery were 18 days longer in 2019/20 than in 2016/17 [1].

Evidence on the value of surgery for both conditions is evolving. For adults with recurrent acute tonsillitis, the NATTINA trial, published in 2023, concluded that surgery is more effective and cost-effective than conservative management [33]. This could result in increases in tonsillectomy, although resource limitations still exist. Two RCTs of DCR may also change practice in the near future [17, 34, 35]. We are not aware of other quantitative studies that have captured the impact of surgical de-implementation on primary care. Other work examining the spillover effects of financial incentives to shift towards day case surgery in English hospitals, similarly found evidence of both positive and negative spillover effects [36].

Our previous work demonstrated that tonsillectomy and Dupuytren’s contracture procedure numbers were falling before the publication of EBI guidelines [7]. Little evidence of any causal impact of the EBI guidelines on trends for either tonsillectomy or DCR procedures were found [7]. Consequently, the spillovers observed here, highlight system-level consequences that accompany de-implementation efforts, regardless of the cause. Therefore, this work offers important considerations for future de-implementation efforts, but should not be interpreted as spillovers resulting directly from the EBI programme itself. De-implementation efforts that successfully reduce surgery, should therefore ensure spillovers are captured, and that primary care is sufficiently resourced to deal with these spillover effects.

### Strengths and limitations

This study utilised high quality data from a large and representative sample of the English population with symptoms of tonsillitis or Dupuytren’s contracture [24, 37]. Therefore, results presented here could reasonably be generalisable to the English population as a whole. Linkage between primary and nationwide secondary care records allowed us to examine patient care from the point of presentation to a GP with symptoms, through specialist referral and on to surgery, if it occurs. CPRD primary care data has several levels of validation and quality assurance before use in research [37]. General Practices have used electronic health records for many years to routinely schedule appointments, record symptoms and prescriptions. For secondary care data, the recording of surgical procedures and outpatient appointments is linked to reimbursement for hospitals, so data quality is generally good [25]. However, clinical coding is not perfect and data on healthcare use may be missing (e.g. if the GP practice stops submitting data to CPRD, the patient leaves the practice, or the patient seeks private healthcare). We have no reason to believe this would happen in significant numbers, or more or less often in the two cohorts. Therefore, we expect minimal bias to be introduced to our results.

The average follow-up period was shorter than proposed due to the impact of COVID-19 on both primary and secondary care. In order to minimise bias, we excluded data after 29th February 2020 when elective surgery was restricted due to the impending national lockdown. We were unable to utilise a control group condition in which a related surgical treatment was not targeted by the EBI programme or not experiencing de-implementation over time. Therefore, we were unable to control in our models for broad changes in health care utilisation trends over time highlighted above. However, our interpretation of the likely impacts are discussed in detail above.

### Implications for policymakers

Any nationwide de-implementation programme is likely to result in spillover effects, both intended and unintended. Indeed, one of the explicit aims of any de-implementation programme is to “free up valuable resources so they can be put to better use elsewhere” [9]. However, de-implementation policies need careful design in order to avoid unintended negative spillovers for NHS staff, patients directly affected by the policies, or patients elsewhere in the system. The aim of de-implementation is rarely to reduce overall spending on healthcare within a governmental or private health care budget. Therefore, it is important to understand how resources are re-allocated within the health system and ensure that they are used for more cost-effective treatment rather than reallocated to similarly low-value or ineffective care.

One potential negative consequence of a de-implementation policy, such as that for tonsillitis, that defines the threshold for surgery based on the number of documented episodes is that patients or parents are incentivised to re-consult rather than self-manage recurrent episodes; similar behaviour has been observed elsewhere [38]. Furthermore, if policies are not sufficiently disseminated or integrated into primary care practice, they may result in patients being referred too early only to be returned to primary care because they do not meet national criteria or too late to the detriment of patient quality of life and the effectiveness of the surgery. One potential positive consequence of a nationwide de-implementation policy is that surgical resources can be reserved for patients most in need, thereby reducing or at least slowing the increases seen in surgical waiting times in England.

### Future research

The work presented here is part of the OLIVIA project [14] which uses mixed methods to investigate the delivery, impact, and acceptability of the EBI programme across England, with a view to producing recommendations to guide future de-adoption programmes. Patients’ perspectives and experiences of seeking care post-publication of EBI guidelines, and the influence of COVID-19 are forthcoming. Healthcare professionals’ accounts of whether and how EBI polices affected their practice will also be reported soon. Further understanding of what happens to health care resources following reductions in surgery across primary and secondary care is important to fully assess the effectiveness of de-adoption policies. These may include impacts on environment and sustainability, surgical reallocation of resources and inequities in access to healthcare resulting from de-implementation.

As discussed elsewhere [7, 15], there is little evidence that the first wave of EBI guidelines which included tonsillectomy and DCR had any substantial impact on pre-existing trends in procedure rates. This is likely to be due, in part, to the fact that most healthcare commissioners in England already had long standing surgical access policies in place, sometimes with more restrictive criteria than the EBI policy. Future analyses of policies to de-implement surgical procedures which result in more rapid de-implementation may show more pronounced spillover effects.

## Conclusions

De-implementation of low-value surgery results in the reallocation of healthcare resources. Here we have highlighted areas where the reduction in low-value surgery have coincided with increases in alternative health care. It is not clear if this reallocation of resources resulted in more cost-effective care. Policy makers therefore should recognise the potential spillover effects of de-implementation at the outset, design policies to mitigate any perceived negative effects, actively promote the cost-effective reallocation of de-implemented resources and monitor spillover effects alongside the impact on the targeted procedure rates.

## Supplementary Information

Supplementary Material 1.

Supplementary Material 2.

## Data Availability

The primary and secondary care data for this project was provided by Clinical Practice Research Datalink (CPRD) [39]. Data was extracted for both CPRD Aurum and Hospital episode statistics (APC and Outpatient) through a single study data sharing agreement between The Sectary of State for Health and Social Care and the University of Bristol. Protocol 22_001729. The data is not owned or held by the authors or their organisation, as such data cannot be made publicly available. An identical extract of the data can be requested from CPRD using the code lists provided in the supplementary materials.

## Abbreviations

EBI (programme): Evidence Based Interventions
NHS: National Health Service
GP: General Practice or General Practitioner.
ICB: Integrated care boards
CCG: Clinical Commissioning Groups
DCR: Dupuytren’s Contracture Release
HES: Hospital Episode Statistics (data source)
CPRD: Clinical Practice Research Datalink (data source)
IMD: Index of multiple deprivation
