# Supplementary material for: Exploring spillover effects following surgical de-implementation: an observational evaluation of primary care, referrals, and time to surgical intervention following reductions in the use of tonsillectomy and Dupuytren’s contracture
Source: Implement Sci Commun. 2026 Feb 12;7:49. doi: 10.1186/s43058-026-00857-7 (PMC13011460; doi:10.1186/s43058-026-00857-7)

# Exploring spillover effects following surgical de-implementation. An observational evaluation of primary care, referrals, and time to surgical intervention following reductions in the use of tonsillectomy and Dupuytren’s Contracture.

## Joel Glynn^1*^; Timothy Jones^2^; Sam Creavin^1^; Carmel Conefrey^1^; Jenny Falco^9^; Nicola Farrar^1^; Mike Bell^3^; Jane Blazeby^1,4,5^; Christopher Burton^6^; Jenny L. Donovan^1^; Andy Gibson^4,7^; Angus McNair^1,4^; Josie Morley^1^; Amanda Owen-Smith^1^; Ellen Rule^8^; Gail Thornton^3^; Victoria Tucker^9^; Iestyn Williams^10^; William Hollingworth^1**^; Leila Rooshenas^1,4,5**^

## Supplementary Tables

***Table S1: Complete case results from part one of the two-part model assessing the likelihood of surgery for both procedures, tonsillectomy and DCR.***

|  | Tonsillectomy Odds Ratio (95% CI), n=336,875 | DCR Odds Ratio (95% CI), n=20,767 |
| --- | --- | --- |
| Cohort |  |  |
| *One (2016/17)* | 1 | 1 |
| *Two (2019/20)* | 0.77 (0.73, 0.82) | 0.88 (0.81, 0.95) |
| Sex |  |  |
| *male* | 1 | 1 |
| *female* | 1.09 (1.03, 1.15) | 0.58 (0.53, 0.64) |
| Age (years) | 1.02 (1.02, 1.02) | 0.99 (0.98, 0.99) |
| GP Consultations (last year) |  |  |
| *0* | 1 | 1 |
| *1* | 5.28 (4.92, 5.66) | 3.36 (2.99, 3.78) |
| *2* | 12.23 (11.22, 13.32) | 4.45 (3.63, 5.45) |
| *3+* | 32.20 (29.75, 34.86) | 2.96 (2.26, 3.88) |
| IMD 2019 |  |  |
| *1 - least deprived* | 1 | 1 |
| *2* | 1.14 (1.04, 1.25) | 1.06 (0.95, 1.19) |
| *3* | 1.16 (1.06, 1.28) | 1.09 (0.97, 1.23) |
| *4* | 1.22 (1.12, 1.34) | 1.16 (1.02, 1.32) |
| *5 - most deprived* | 1.25 (1.15, 1.37) | 0.90 (0.77, 1.02) |
| Ethnicity |  |  |
| white | 1 | 1 |
| *Asian* | 0.76 (0.68, 0.85) | 0.43 (0.26, 0.70) |
| *black* | 0.87 (0.74, 1.03) | 0.50 (0.26, 0.96) |
| *mixed* | 1.06 (0.91, 1.23) | 0.50 (0.15, 1.61) |
| *other* | 0.89 (0.75, 1.05) | 0.55 (0.31, 0.97) |
|  |  |  |

***Table S2: Complete case results from part two of the two-part model assessing time-to-surgery in the subset of patients that received either a tonsillectomy or DCR procedure.***

|  | Tonsillectomy Ratio (95% CI), n=5,625 | DCR Ratio (95% CI), n=2,836 |
| --- | --- | --- |
| Cohort |  |  |
| *2016/17* | 1 | 1 |
| *2019/20* | 0.95 (0.92, 0.99) | 0.98 (0.93, 1.03) |
| Sex |  |  |
| *male* | 1 | 1 |
| *female* | 1.03 (0.99, 1.07) | 0.99 (0.93, 1.05) |
| Age (years) | 1.00 (1.00, 1.01) | 1.00 (1.00, 1.00) |
| GP Consultations (last year) |  |  |
| *0* | 1 | 1 |
| *1* | 1.08 (1.03, 1.13) | 0.75 (0.70, 0.80) |
| *2* | 1.04 (0.98, 1.10) | 0.58 (0.52, 0.64) |
| *3+* | 0.97 (0.92, 1.01) | 0.56 (0.48, 0.65) |
| IMD 2019 |  |  |
| *1 - least deprived* | 1 | 1 |
| *2* | 1.01 (0.95, 1.07) | 0.99 (0.93, 1.07) |
| *3* | 0.98 (0.92, 1.04) | 0.98 (0.91, 1.06) |
| *4* | 0.98 (0.92, 1.04) | 0.96 (0.89, 1.04) |
| *5 - most deprived* | 1.00 (0.94, 1.05) | 1.03 (0.94, 1.13) |
| Ethnicity |  |  |
| white | 1 | 1 |
| *Asian* | 0.97 (0.91, 1.04) | 0.99 (0.72, 1.37) |
| *black* | 0.98 (0.88, 1.09) | 0.54 (0.35, 0.82) |
| *mixed* | 0.98 (0.89, 1.08) | 1.31 (0.61, 2.80) |
| *other* | 0.99 (0.89, 1.11) | 1.02 (0.71, 1.47) |

## Medical Codes


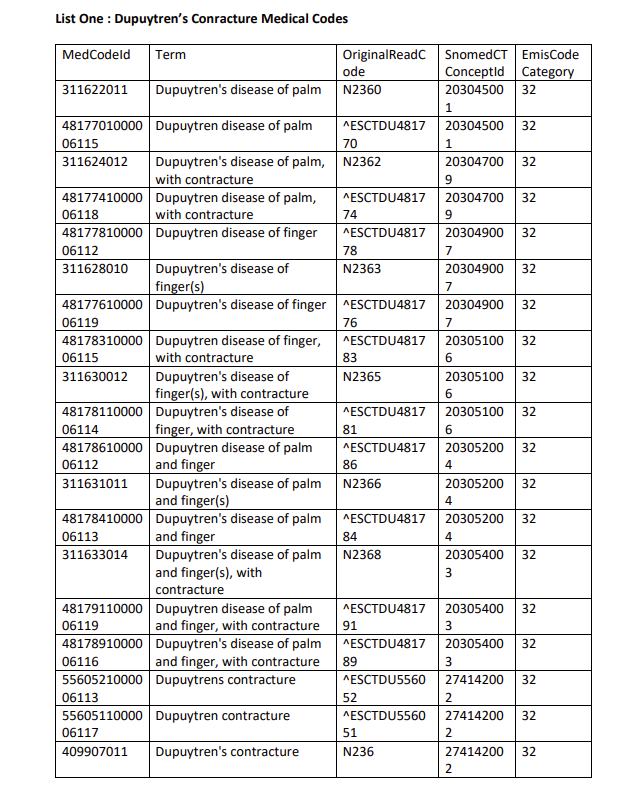


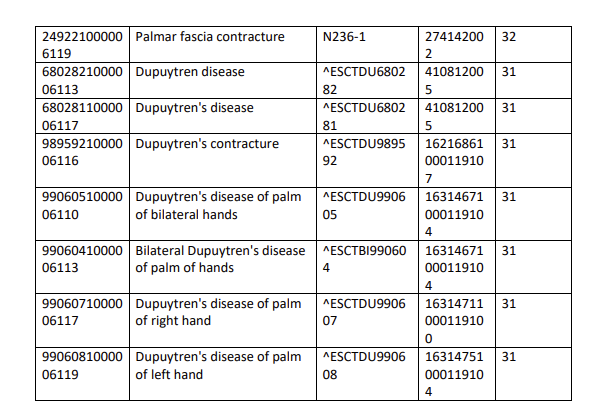


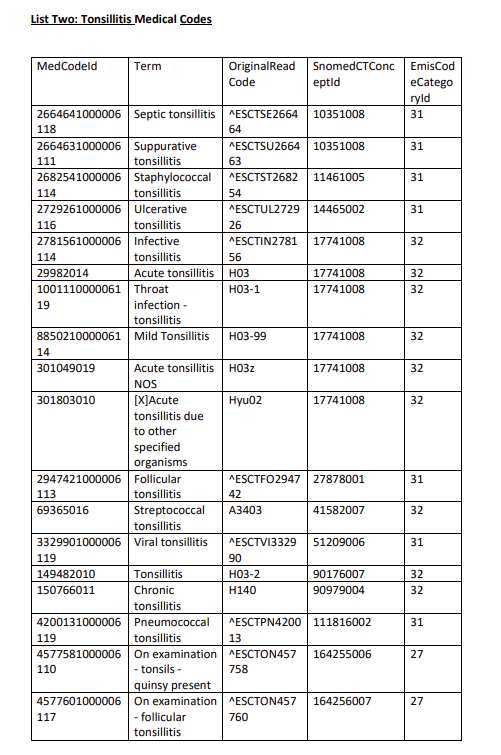


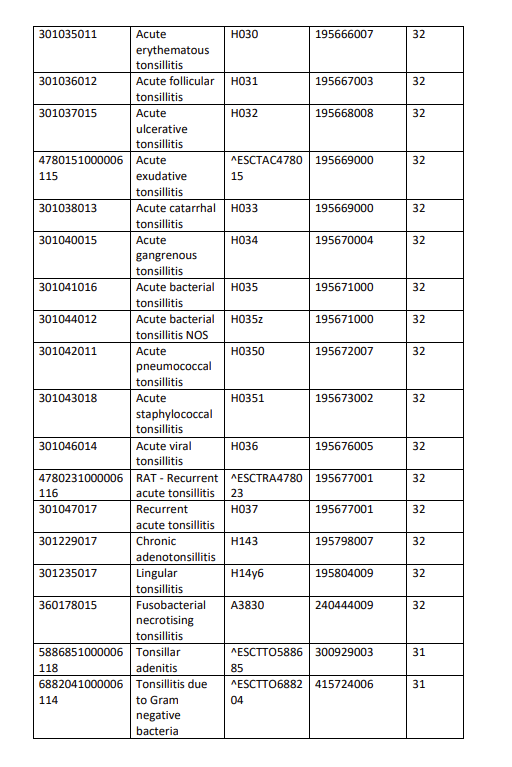


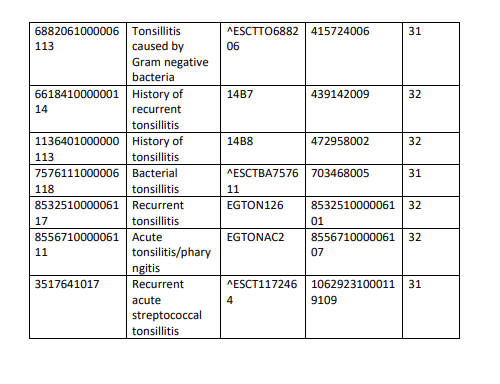

Supplement: Supplementary file 1 — Supplementary Material 1. [file 43058_2026_857_MOESM1_ESM.docx]
